# Bogus Diplomas

**Published:** 1884-05

**Authors:** 


### BOGUS DIPLOMAS.

England is being flooded with fraudulent diplomas, purporting to be issued by "The Wilmington, Del., Dental College." Of course there is no such institution. How long, O Lord! how long shall American dentistry rest under the opprobrium and shame of seeing spurious diplomas hawked about Europe, while we at home are powerless to prevent it?

